# The Doctrine of Signatures

**Published:** 1909-04-24

**Authors:** 


### Medical Antiquities.

### THE DOCTRINE OF SIGNATURES.

It would appear to be one of primitive man's inherent beliefs that casual connection in thought equals causal relation in fact—the most infrequent of coincidences, analogies utterly superficial or wholly subjective, strike him as having great practical significance. He constantly confuses subject with object, too, and for an explanation of physical facts nearly always turns to the supernatural. The truth of this quartette of anthropological commonplaces is seldom better shown than in early medicine. When Apollonius of Tyana treated delay in labour by making the husband carry a hare round and round the patient, he may well have been acting in good faith and with fair expectation of success. Reasoning on the same lines, a student of Æsop might have amended the treatment by substituting a tortoise, reserving the former animal rather for a case in which danger was threatening the perineum. Again, those who gave the name "eye-bright" to a certain plant earnestly believed that the black pupil-like spot in its corolla was placed there by a benevolent Providence as an indication for use in diseases of the eye. The name of this curious belief in therapeutic symbols is the doctrine of signatures. Obviously, considering the complexity of nature, its followers had the advantage of a large pharmacopœia; no doubt at all, too, they could point to some good results. Yellow turmeric might fail of an immediate effect on jaundice, but probably before many other non-poisonous and not too outrageously unpalatable substances of the right hue had been tried, catarrh of the bile-ducts would have yielded to the inexpugnable *vis medicatrix* of long-suffering nature. We know now that hæmorrhage of itself makes the remaining blood more coagulable, and so are in no doubt as to how the bloodstone stopped bleeding. Walnuts are rather like the cerebral convolutions to look at, and possibly, even among the not very distant descendants of the precibiculturists, may also have been a good diet for lunatics. An often quoted example of treatment after the doctrine of signatures is that of John of Gaddesden, who claimed to have recovered Edward II. of smallpox by wrapping him in red flannel. With the same object of bringing out the rash red substances, like rose-leaves, were mingled with the patient's drink. This recalls the highly unpoetical comparisons in Dryden's early lines on the death of Lord Hastings from smallpox.

> ". . . which through's flesh did sprout
> Like rosebuds, stuck 'i the lily skin about."

The fallacy of reasoning underlying all these grotesqueries is hardly extinct yet, an instance being in evidence about the time that tomatoes were becoming a common article of diet in this country. A tomato when cut resembles rather the section of a carcinoma, and so the one was rumoured to be the cause of the other. Tomatoes and cancer is, of course, not quite on all-fours with walnuts and lunacy, but still even here the homœopathists might have risen to the occasion. Happily medicine has ceased to have anything to do with metaphysical vapourings, but nevertheless it should make better acquaintance yet with logic. In a common disease like consumption (the natural intermissions of which have made many a quack's fortune) the list of measures which have "done good," whilst regrettably long, is still being added to. As Whistler caustically remarked, a man may be misled by "an overwhelming conviction of right"; and all the more easily if he has been encouraged to entertain a good opinion of himself. The *ipsi dixit* of eminence is no substitute for rational proof: in his own day and country, there can have been no more eminent clinician than John of Gaddesden.

